# Unterschiede zwischen Rettungsdiensteinsätzen mit und ohne Patiententransport: Eine retrospektive Analyse der Leitstellendaten in einem Flächenstaat

**DOI:** 10.1007/s00103-022-03590-3

**Published:** 2022-09-16

**Authors:** Florian Dax, Heiko Trentzsch, Marc Lazarovici, Kathrin Hegenberg, Katharina Kneißl, Florian Hoffmann, Stephan Prückner

**Affiliations:** 1 Institut für Notfallmedizin und Medizinmanagement (INM), Klinikum der Universität München, LMU München, Schillerstr. 53, 80336 München, Deutschland; 2 Dr. von Haunersches Kinderspital, Kinderklinik und Kinderpoliklinik, Klinikum der Universität München, LMU München, München, Deutschland; 3 Landesgeschäftsstelle, Bayerisches Rotes Kreuz (BRK), München, Deutschland

**Keywords:** Integrierte Leitstelle, Einsatzgrund, Patientenversorgung, Notfalleinsatz, Qualitätsmanagement, Integrated dispatch center, Alarm keyword, On-site treatment, Emergency response, Quality management

## Abstract

**Hintergrund:**

Die Inanspruchnahme des Rettungsdienstes in Bayern steigt seit Jahren an. Wir haben die Hypothese aufgestellt, dass Notfalleinsätze ohne Patiententransport (RoT) häufig Ausdruck einer unzureichenden Alarmierungsplanung sind. Das Ziel der Studie war es, für solche Einsätze die Unterschiede zwischen den Integrierten Leitstellen (ILS) in Bezug auf die Merkmale Transportquoten und Spannweiten nach Einsatzgrund sowie Uhrzeiten und Wochentage zu beschreiben.

**Methode:**

Retrospektive Querschnittstudie der Daten aller 26 ILS des Freistaats Bayern im Jahr 2018. Transportquoten für wesentliche Einsatzgründe bei Notfalleinsätzen ohne Notarztbeteiligung wurden in Abhängigkeit von Leitstellenbereich, Tageszeit und Wochentag vergleichend analysiert. Einsätze wurden kategorisiert als RoT oder TP (Rettungswageneinsatz mit Transport).

**Ergebnisse:**

Von 510.145 Einsätzen waren 147.621 (28,9 %) RoT und 362.524 (71,1 %) TP. Für alle untersuchten Einsatzgründe zeigten sich deutliche regionale Unterschiede in der Transportquote. Die höchste Spannweite unter den ILS ergab sich für die Einsatzgründe „Brandmeldeanlage“ (16,8 Prozentpunkte), „Hausnotruf aktiver Alarm“ (16,1) sowie „Herz/Kreislauf“ (14,6). In den Morgenstunden sinkt das Einsatzaufkommen bei steigenden TP. Die wenigsten RoT fanden zwischen 8 und 10 Uhr statt. Die Analyse der Wochentage ergab kleine Unterschiede in der Häufigkeit von RoT an Montagen sowie an Wochenenden ohne planerische Relevanz.

**Schlussfolgerung:**

Wir haben deutliche Unterschiede in den Spannweiten festgestellt. Dies könnte auf örtlich unterschiedliche Alarmierungsplanungsvorgaben oder Dispositionsentscheidungen der ILS hindeuten. Die Leitstellen weisen hier wahrscheinlich ein erhebliches Potenzial zur Steuerung und Verbesserung der Ressourcenallokation auf.

**Zusatzmaterial online:**

Zusätzliche Informationen sind in der Online-Version dieses Artikels (10.1007/s00103-022-03590-3) enthalten.

## Abstract (en)

**Background:**

The use of the emergency medical services (EMS) in Bavaria has been increasing for years. We hypothesized that emergency response without patient transport (RoT) is often an expression of inadequate alert planning. The aim of the study was to describe the differences between the integrated dispatch centers (ILSs) for such operations with regard to the characteristics of transport quotas and ranges according to the reason for deployment as well as times and days of the week.

**Method:**

Retrospective cross-sectional study of data from all 26 ILSs in the Free State of Bavaria in 2018. Transport quotas for emergency operations for essential reasons without emergency physician involvement were analyzed comparatively in relation to dispatch center area, time of day, and day of the week. Deployments were categorized as RoT or ambulance deployment with transport (TP).

**Results:**

Of 510,145 call-outs, 147,621 (28.9%) were RoT and 362,524 (71.1%) were TP. There were significant regional differences in the transport quotas for all deployment reasons investigated. The highest range among the ILSs was found for the deployment reasons “fire alarm system” (16.8 percentage points), “personal emergency response system” (16.1%), and “heart/circulation” (14.6%). In the morning hours, the number of calls decreases with increasing TP. The fewest RoT took place between 8 and 10 am. The days of the week analysis revealed small differences in the frequency of RoT on Mondays as well as on weekends without planning relevance.

**Conclusion:**

We found significant differences in the ranges. This could indicate locally different alert planning specifications or dispatching decisions by the ILS. The control centers probably have considerable potential for controlling and improving resource allocation.

## Einleitung

Im Freistaat Bayern stieg die Vorhaltung von Rettungswagen (RTW) von insgesamt 3.328.900 h im Jahr 2010 auf 3.958.900 h im Jahr 2020 an (+ 18,9 %; [1, 2]). Im selben Zeitraum zeigte sich ein Anstieg der Notfallereignisse um 37 %, von 745.600 auf 1.024.500 Ereignisse [1, 2]. Diese Steigerungen wurden sowohl im städtischen als auch im ländlichen Raum beobachtet [3]. Nach Auffassung der Autoren werden aus Kostengründen Konzepte benötigt, um die stetige Zunahme an Rettungsmitteln aufgrund zunehmender Einsatzzahlen zu stoppen.

Ein Potenzial zur Reduktion von Fehlallokationen bieten Rettungswageneinsätze ohne Transport von Patienten (RoT). Möglicherweise müssen diese Einsätze nicht zwingend mit einem RTW bedient werden. Durch eine Einsparung von Rettungsmitteln bei RoT könnten mehr Ressourcen für Rettungstransporte bereitgestellt werden. Wir haben die Hypothese aufgestellt, dass RoT häufig Ausdruck einer unzureichenden Alarmierungsplanung sind. Lokale Unterschiede bei bestimmten Einsatzgründen könnten ein Hinweis auf Unterschiede in der örtlich durchgeführten Alarmierungsplanung sein und damit Hinweise auf bestehende Optimierungsmöglichkeiten bei der Ressourcenplanung, -gewichtung und -allokation geben.

Uns sind bisher keine Arbeiten bekannt, die zeigen, dass es in Abhängigkeit vom Einsatzgrund vermehrt zu einem RoT kommt. Das Ziel der Studie war es, Unterschiede von RoT-Einsätzen zwischen den Leitstellen in Bezug auf die Merkmale Transportquoten und Spannweiten nach Einsatzgrund sowie Uhrzeiten und Wochentagen zu beschreiben. Die Analyse erfolgte ausschließlich im Hinblick auf die Inanspruchnahme der öffentlich-rechtlichen rettungsdienstlichen Komponente. Die Arbeit ist Bestandteil der Studie „Rettungswageneinsatz ohne Transport (RoT)“. Die Beauftragung zur Durchführung erfolgte durch das Bayerische Staatsministerium des Innern, für Sport und Integration (BayStMI).

### Alarmierung des Rettungsdienstes und Datenerhebung in Bayern

Der Freistaat Bayern ist in 26 Rettungsdienstbereiche (RDB) gegliedert [4]. Die Landkreise und kreisfreien Gemeinden haben – organisiert in Zweckverbänden für Rettungsdienst und Feuerwehralarmierung (ZRF) – die Aufgabe, Notfallrettung und Krankentransport im übertragenen Wirkungskreis flächendeckend sicherzustellen [5], und sind für die Alarmierungsplanungen zuständig [6]. In jedem Rettungsdienstbereich müssen eine Integrierte Leitstelle (ILS), ein Ärztlicher Leiter Rettungsdienst (ÄLRD) sowie ganztägig einsatzbereite Rettungswachen und Notarztstandorte vorhanden sein [7].

Die ILS sind flächendeckend unter der europäischen Notrufnummer 112 erreichbar und koordinieren sämtliche Einsätze der Notfallrettung. Die Notrufabfrage erfolgt nichtstandardisiert auf Basis der Empfehlung zur strukturierten Notrufabfrage des Rettungsdienstausschusses Bayern [8]. Für die strukturierte Notrufabfrage steht den Einsatzsachbearbeitern (Disponenten) keine Softwareunterstützung zur Verfügung. Der Einsatzgrund als Ergebnis der Notrufabfrage ergibt sich aus den bayernweit identischen Einsatzschlagwörtern der Alarmierungsbekanntmachung [6].

Zur Einsatzerfassung, -disposition und -begleitung wurde durch den Freistaat Bayern für sämtliche ILS im Rahmen einer Landeslizenz ein einheitliches Einsatzleitsystem beschafft und zur verpflichtenden Nutzung bereitgestellt [9]. Die Ausbildung zum Disponenten ILS ist in Bayern staatlich geregelt: Neben einer rettungsdienstlichen sowie feuerwehrfachlichen Qualifikation sieht die Verordnung einen mehrwöchigen Disponentenlehrgang an der Staatlichen Feuerwehrschule Geretsried vor [10]. Über das Tetra-Digitalfunknetz können die Einsatzkräfte Statusmeldungen an die ILS übermitteln (wesentliche Statusmeldungen: 1 = einsatzbereit über Funk, 3 = Einsatz übernommen, 4 = Einsatzstelle an, 7 = Patient aufgenommen, 8 = Zielort an).

Die im Einsatzleitsystem der ILS in Bayern beschriebenen Einsatzgründe wurden durch das Bayerische Staatsministerium des Innern, für Bau und Verkehr einheitlich festgelegt [6]. Die in der Anlage 1 dieser „Alarmierungsbekanntmachung“ aufgeführten Zustandsbeschreibungen geben dem Disponenten Hinweise auf die für die Alarmierung zu verwendenden Schlagwörter. Für die Rettungsdienstalarmierung stehen den ILS in Bayern insgesamt 111 Einsatzschlagwörter zur Verfügung [6]. Diese Schlagwörter stellen gleichzeitig den Einsatzgrund für den Rettungsdienst dar und werden von der Leitstelle an die Fahrzeugbesatzungen übermittelt.

Das Institut für Notfallmedizin und Medizinmanagement (INM) wurde durch das BayStMI mit der Datenauswertung der Leitstellendaten zum rettungsdienstlichen Einsatzgeschehen für den gesamten Freistaat beauftragt [11]. Auf dieser rechtlichen Grundlage werden dem INM in regelmäßigen und definierten Abständen die Daten in anonymisierter und standardisierter Form übermittelt. Diese Datenbasis bietet eine Vielzahl von Untersuchungs- und Forschungsmöglichkeiten und kann für Forschungszwecke verwendet werden. Die Beauftragung zur Durchführung der Studie RoT erfolgte durch das BayStMI als separater Projektauftrag, damit zusätzliche Wertschöpfung aus dem Datenbestand generiert und mit den Ergebnissen möglicherweise der erhöhten Nachfrage nach Rettungsdienstressourcen entgegengewirkt werden kann. Die Datensätze enthalten Informationen zu Einsatzgrund und Vor-Ort-Zeit.

## Material und Methodik

### Datenquelle und Stichprobe

Für die vorliegende quantitative Analyse wurden die aus den ILS Bayerns an das INM übermittelten Daten vom 01.01.2018–31.12.2018 herangezogen.

**Figure 1:**
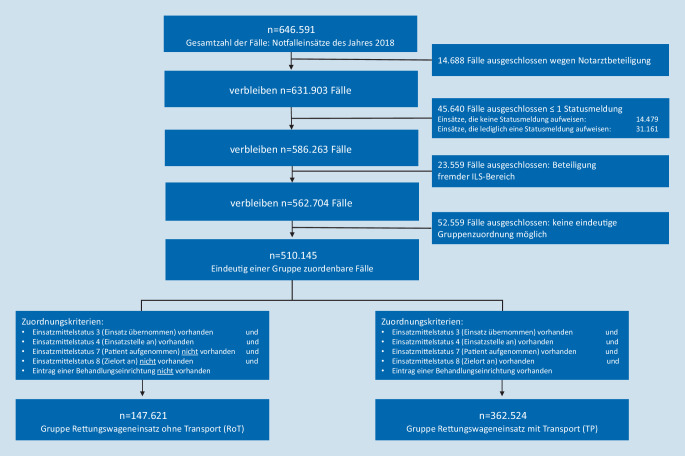

Zur Beantwortung der Forschungsfrage wurden 2 Gruppen (Rettungswageneinsatz ohne Transport [„RoT“] und Rettungswageneinsatz mit Transport [„TP“]) gebildet. Die Zuordnungskriterien zu den Gruppen bildeten sowohl die Statusmeldungen als auch die Dokumentation zu Behandlungseinrichtungen (Abb. 1). Es wurden nur Einsätze ohne Notarztbeteiligung eingeschlossen, da diese möglicherweise zumindest anteilig auch mit alternativen Einsatzmitteln abgewickelt werden können. Ausgeschlossen waren Fälle, die über keine oder lediglich eine einzige Statusmeldung des Funkmeldesystems (FMS) verfügen. Hier muss davon ausgegangen werden, dass das Einsatzmittel die Einsatzstelle nicht erreicht hat oder es sich um von Einsätzen abgezogene RTW oder Testeinsätze handelte. Ausgeschlossen wurden zudem systembedingte Einsatzkopien bei bereichsübergreifenden Einsätzen („fremde Leitstellenbereiche“) sowie Fälle, die aufgrund fehlender Plausibilitäten nicht eindeutig einer der beiden Gruppe zugewiesen werden konnten. Für Ereignisse, bei denen der Disponent anstelle eines Zielorts vermerkt hatte, dass der Patient nicht transportiert wurde, wurde der Fall manuell der RoT-Gruppe zugewiesen.

Zur Auswertung der Einsatzgründe wurden sämtliche verfügbare Daten der Leitstellen herangezogen. Es wurden nur Einsatzgründe mit mindestens 1000 Einsätzen untersucht. Deren Definitionen sind dem Onlinematerial zu entnehmen.

### Statistische Methoden

Die Gruppen RoT und TP wurden miteinander verglichen. Anhand der Summe der beiden Gruppen wurde die prozentuale Häufigkeit RoT und TP berechnet. Zur Beantwortung der Forschungsfrage, ob bei RoT-Einsätzen Unterschiede zwischen den Leitstellen in Bayern existieren, erfolgte die Berechnung der prozentualen RoT- bzw. Transportquoten für alle 26 bayerischen Leitstellenbereiche je Einsatzgrund. Somit können für jeden Einsatzgrund ein Mittelwert, die Standardabweichung sowie die Spannweite angegeben werden. Die Spannweite errechnet sich als Differenz zwischen Maximal- und Minimalwert. Sie ist der Indikator für örtliche Unterschiede.

Zur Analyse der Anteile von Rot und TP zu bestimmten Uhrzeiten des Einsatzbeginns wurde eine Häufigkeitssortierung nach der Stunde des Einsatzbeginns durchgeführt. Zur Überprüfung signifikanter Unterschiede wurde innerhalb der beiden Gruppen RoT und TP ein Test auf Binomialverteilung bei einem Signifikanzniveau von 0,05 vorgenommen. Die Nullhypothese lautete: Die RoT-Einsätze sind zu allen Uhrzeiten gleich verteilt. Als Referenzwert diente das arithmetische Mittel der prozentualen Anteile der Gesamtstudienpopulation (RoT = 0,29, TP = 0,71).

Die Anteile von Rot und TP an den Wochentagen (nach Einsatzbeginn) wurden sowohl deskriptiv analysiert und in einem Balkendiagramm dargestellt als auch statistisch mittels Chi^2^-Test vergleichend analysiert.

Zur Datenanalyse wurden Microsoft Excel 2016 (Microsoft, Redmond, WA, USA), IBM SPSS Statistics 26 (IBM, Armonk, NY, USA) und R‑4.0.3/RStudio 1.3 (RStudio,PBC, Boston, MA, USA) verwendet.

## Ergebnisse

In die Analyse wurden insgesamt 510.145 Fälle eingeschlossen. Davon entfielen 147.621 Einsätze auf die Gruppe RoT (29 %) und 362.524 auf die Gruppe TP (71 %).

### Unterschiede bei den Einsatzgründen zwischen den Leitstellenbereichen.

Die Tab. 1 zeigt für die 18 Einsatzgründe mit mindestens 1000 Einsatzereignissen (= 99,8 % der gesamten Fälle) die Anteile von RoT-Einsätzen für den jeweiligen Leitstellenbereich. Die höchste Spannweite ergab sich bei den Einsatzgründen „Brandmeldeanlage“ (16,8 Prozentpunkte), „Hausnotruf aktiver Alarm“ (16,1), „Herz/Kreislauf“ (14,6), „Brand mit RD“ (11,8, RD: Rettungsdienst) sowie „Trauma“ (10,0). In Krumbach (ILS Donau-Iller) waren 16,8 % aller RoT-Einsätze auf den Einsatzgrund „Brandmeldeanlage“ zurückzuführen, in den ILS Coburg und ILS Traunstein 0,0 %. Beim Einsatzgrund „Hausnotruf aktiver Alarm“ erhielt die ILS den Einsatz durch Vermittlung einer Hausnotrufzentrale einer Hilfsorganisation oder eines privaten Betreibers. In der ILS Coburg entfielen 18,0 % der RoT-Einsätze auf diesen Einsatzgrund, in Erding 1,9 %. Der Einsatzgrund „Herz/Kreislauf“ wies in den ILS Erding und Aschaffenburg 25 % Anteil an RoT-Einsätzen auf, in der ILS Coburg 10,4 %. Die niedrigste Spannweite und damit die geringsten Unterschiede zwischen den Leitstellen konnten bei den Einsatzgründen „Geburt“ und „Psych“ festgestellt werden.

**Table 1:** 

Einsatzgrund	Amberg	Ansbach	Aschaffenburg (Bayer. Untermain)	Augsburg	Bamberg-Forchheim	Bayreuth/Kulmbach	Coburg	Erding	Fürstenfeldbruck	Hof (Hochfranken)	Kempten (Allgäu)	Krumbach (Donau-Iller)	Landshut	München	Nürnberg	Passau	Regensburg	Region Ingolstadt	Rosenheim	Schwabach (Mittelfranken-Süd)	Schweinfurt	Straubing	Traunstein	Weiden (Nordoberpfalz)	Weilheim (Oberland)	Würzburg	*Spannweite (Prozentpunkte)*	*Mittelwert*	*Standardabweichung um den Mittelwert*
BMA (Brandmeldeanlage)	1,7	7,2	0,4	6,1	11,5	15,5	0,0	0,3	9,0	11,1	8,8	16,8	9,3	0,2	8,1	0,1	0,6	13,6	5,3	7,4	10,5	0,2	0,0	15,0	15,9	6,4	*16,8*	*7,0*	*5,6*
Hausnotruf aktiver Alarm	7,1	6,0	2,4	2,4	11,1	12,9	18,0	1,9	3,7	12,2	10,5	3,5	5,2	2,3	2,8	4,2	5,4	2,5	4,9	7,2	10,4	7,0	7,0	7,4	6,3	6,0	*16,1*	*6,6*	*3,9*
Herz/Kreislauf	13,5	16,7	25,0	17,2	15,2	13,1	10,4	25,0	18,4	11,5	19,3	21,6	16,5	20,6	19,9	21,5	17,5	16,1	19,9	17,6	17,3	15,1	19,3	13,0	11,9	15,2	*14,6*	*17,2*	*3,7*
Brand mit RD (Rettungsdienst) – mit und ohne vitale Bedrohung	12,3	9,4	4,9	5,3	4,6	10,6	5,5	4,0	3,3	6,8	4,0	4,8	6,6	0,5	5,7	7,8	6,2	5,7	4,7	5,1	5,1	6,7	10,2	7,6	2,9	3,9	*11,8*	*5,9*	*2,5*
Trauma	13,1	14,0	12,3	14,1	10,4	8,2	17,6	15,3	15,2	13,5	11,5	8,6	11,0	17,5	12,4	15,4	12,2	12,3	14,5	13,8	11,9	11,6	11,9	12,9	18,2	17,3	*10,0*	*13,3*	*2,5*
Sonstiges Ereignis/Zustand	10,7	11,8	13,1	10,8	9,7	8,2	15,0	14,5	8,9	10,2	9,9	9,7	11,1	9,3	6,4	13,0	7,7	10,5	14,4	13,7	12,1	12,7	8,6	9,9	14,1	13,5	*8,6*	*11,1*	*2,3*
Intoxikation	4,9	5,4	5,7	6,9	4,3	2,4	4,8	3,4	3,6	3,1	3,8	3,5	4,6	8,6	8,1	5,4	10,3	4,9	3,2	3,6	3,2	9,2	2,9	4,9	1,9	4,2	*8,4*	*4,9*	*2,1*
Trauma – Verkehrsunfall (VU) nur RD	8,5	4,7	6,7	3,7	6,9	5,7	4,2	6,7	4,1	3,9	3,9	4,3	8,2	3,5	4,0	9,1	9,3	6,0	6,8	5,7	5,6	11,6	11,5	7,2	5,1	3,8	*8,1*	*6,2*	*2,3*
Bewusstsein	3,4	3,1	2,6	4,1	4,3	4,3	3,6	1,4	2,5	3,5	2,9	2,5	1,7	8,7	4,6	0,9	4,0	4,2	1,2	2,8	2,1	2,1	2,5	3,0	2,0	5,9	*7,8*	*3,2*	*1,6*
Technische Hilfeleistung (THL) mit RD	5,2	2,7	3,5	6,4	5,1	5,4	5,9	2,1	3,1	5,9	4,2	4,1	3,0	4,6	6,8	4,5	8,7	4,2	4,8	3,5	4,1	2,0	6,5	2,5	3,6	3,4	*6,7*	*4,5*	*1,6*
Kind (bis 12 Jahre) Trauma	1,8	1,4	2,0	2,5	1,2	1,6	1,3	5,5	4,6	1,2	1,8	1,6	2,3	2,9	1,7	1,5	2,2	2,0	2,5	3,0	1,4	2,3	2,6	0,9	2,6	1,5	*4,6*	*2,2*	*1,0*
Schmerzen	2,2	3,5	3,0	4,7	2,3	1,2	2,4	4,7	4,7	2,9	2,9	3,0	3,2	5,1	3,7	2,3	1,9	3,1	3,3	2,5	2,4	1,8	1,4	1,8	2,8	4,9	*3,9*	*3,0*	*1,1*
Ärger	2,2	0,9	2,1	2,1	1,9	0,9	1,4	1,3	1,3	2,5	2,0	1,7	2,1	1,1	1,5	2,0	2,7	2,0	1,6	0,8	2,7	4,6	2,2	2,0	1,1	1,1	*3,8*	*1,8*	*0,8*
Neuro	4,1	3,8	5,5	3,5	4,0	3,6	2,2	2,8	4,9	3,5	5,0	3,4	5,1	5,8	4,8	5,0	3,3	3,7	4,3	3,6	3,5	4,0	6,0	3,6	2,9	4,7	*3,8*	*4,1*	*0,9*
Kind (bis 12 Jahre) erkrankt	2,2	2,6	2,0	3,4	2,1	1,6	1,8	4,9	5,1	2,7	2,9	2,8	3,2	3,2	1,9	2,2	2,2	3,1	2,8	3,6	1,9	3,0	1,7	1,6	2,8	1,8	*3,5*	*2,7*	*0,9*
Atmung	5,0	4,9	6,2	5,3	4,2	3,7	4,0	5,0	5,4	3,6	5,2	6,6	3,9	5,1	5,8	3,7	4,2	4,1	4,3	4,2	4,0	3,7	4,1	4,3	4,4	4,6	*3,0*	*4,6*	*0,8*
Psych	0,8	1,3	2,2	1,2	0,8	0,6	0,5	0,9	1,5	1,2	0,8	0,8	1,2	0,6	0,9	1,0	1,2	0,7	0,8	1,2	1,2	1,6	0,7	2,1	1,1	1,3	*1,7*	*1,1*	*0,4*
Geburt/Entbindung	0,0	0,0	0,0	0,0	0,0	0,1	0,0	0,1	0,1	0,0	0,0	0,1	0,0	0,2	0,1	0,0	0,1	0,0	0,0	0,0	0,0	0,1	0,0	0,0	0,1	0,0	*0,2*	*0,0*	*0,1*
*Summe*	*98,7*	*99,4*	*99,6*	*99,7*	*99,6*	*99,6*	*98,6*	*99,8*	*99,4*	*99,3*	99,4	*99,4*	*98,2*	*99,8*	*99,2*	*99,6*	*99,7*	*98,7*	*99,3*	*99,3*	*99,4*	*99,3*	*99,1*	*99,7*	*99,7*	*99,5*	*–*	*–*	*–*

### Anteile der Einsatzarten nach Uhrzeit.

**Figure 2:**
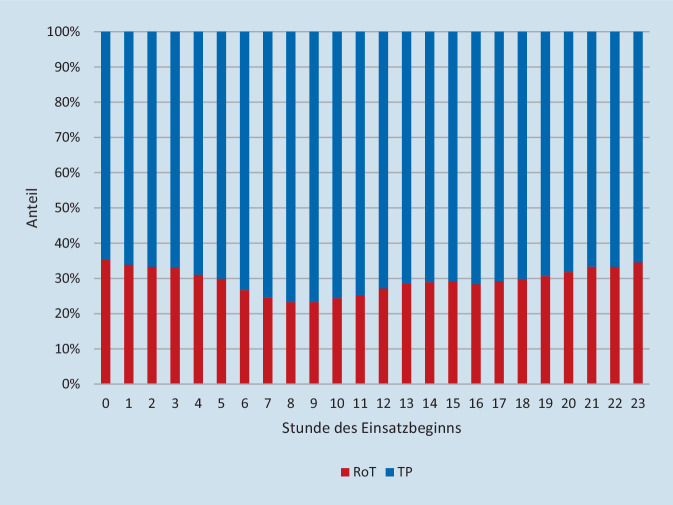

**Figure 3:**
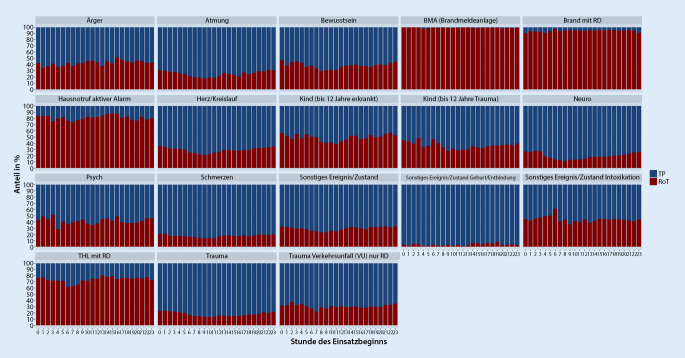

**Figure 4:**
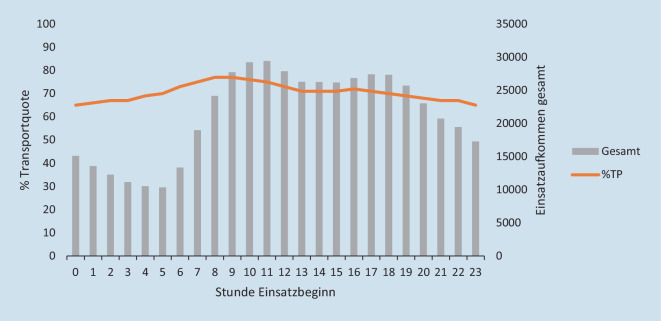

Die deskriptive Betrachtung der Einsatzarten nach Uhrzeit zeigt eine etwas niedrigere RoT-Quote im Zeitraum von 6 bis 12 Uhr (Abb. 2). Es konnten keine tageszeitlichen Besonderheiten bei den einzelnen Einsatzgründen beobachtet werden (Abb. 3). Die größten Abweichungen ergaben sich bei Einsatzbeginn um 8 sowie um 9 Uhr. Im Test auf Binomialverteilung zeigten sich diese beiden Uhrzeiten mit dem größten Unterschied zum arithmetischen Mittel. In diesem Zeitraum fanden prozentual die meisten TP bzw. die wenigsten RoT statt. Keine signifikante Abweichung vom arithmetischen Mittel konnte bei den Uhrzeiten 13 Uhr (*p* = 0,245), 14 Uhr (*p* = 0,935), 15 Uhr (*p* = 0,844) sowie 17 Uhr (*p* = 0,545) festgestellt werden. Zu sämtlichen anderen Uhrzeiten waren die Unterschiede signifikant (um 16 Uhr *p* = 0,022, restliche Uhrzeiten *p* = < 0,001). In Abb. 4 wird die Gesamtzahl der Einsätze der Transportquote gegenübergestellt. Der Grafik können die beschriebenen Abweichungen vom arithmetischen Mittel der Transportquote entnommen werden. Außerdem zeigt die Grafik, dass zwischen 0 und 5 Uhr ein Anstieg der Transportquote bei gleichzeitigem Rückgang der Gesamteinsatzzahlen beobachtet werden kann.

### Anteile der Einsatzarten an Wochentagen.

**Figure 5:**
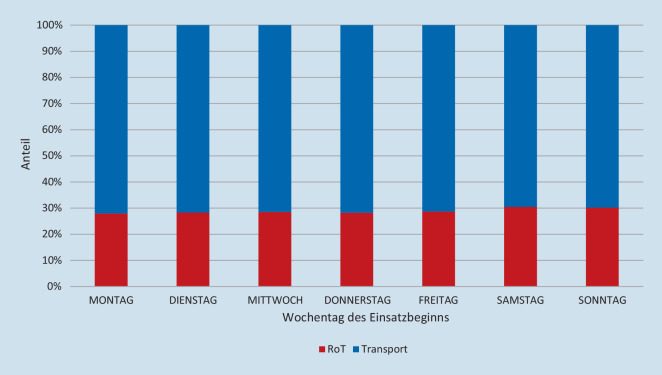

Die deskriptive Betrachtung der Verteilung von RoT-Einsätzen auf die Wochentage lässt keine Aussage über wesentliche Unterschiede zu (Abb. 5). Die statistische Auswertung mittels Chi^2^-Tests zeigt einen signifikanten Unterschied in der Verteilung der RoT-Häufigkeiten (*p* < 0,001). Diese Unterschiede sind darauf zurückzuführen, dass am Montag höhere und am Samstag und Sonntag niedrigere RoT-Quoten als erwartet beobachtet wurden. Dieser Unterschied beträgt zwischen 738 und 823 Einsätzen pro Tag gegenüber einem Mittelwert für die anderen Tage.

## Diskussion

Etwa ein Drittel (29 %) der Rettungsdiensteinsätze in Bayern erfolgt ohne Patiententransport. Für alle untersuchten Einsatzgründe zeigt sich je nach Leitstelle eine mehr oder weniger stark ausgeprägte Variabilität bei der Häufigkeit von RoT-Einsätzen. Besonders ausgeprägt ist dies bei bestimmten Feuerwehreinsatzgründen sowie Einsätzen, die von Hausnotrufzentralen übermittelt werden.

In Bayern existieren 7 Berufsfeuerwehren mit den entsprechenden Strukturen und teilweise eigenen (Zug‑)RTW. In der Fläche Bayerns werden Feuerwehreinsätze durch Freiwillige Feuerwehren ausschließlich unter Inanspruchnahme des öffentlich-rechtlichen Rettungsdienstes oder Zusatzvorhaltungen der Hilfsorganisationen abgewickelt. Bei Berufsfeuerwehren haben die (Zug‑)RTW-Aufgaben als Sicherungstrupp für den Feuerwehreinsatz. RTW der Hilfsorganisationen können diese Aufgaben nicht wahrnehmen, sondern dienen lediglich als rettungsdienstliche Absicherung. Unserer Meinung nach entspricht die Entsendung von öffentlich-rechtlichen RTW zu bestimmten Feuerwehreinsatzgründen nicht der Einsatzanforderung. Ferner muss diskutiert werden, ob durch diese Verwendung andere Notfallereignisse nicht oder nur verzögert bedient werden können. Dies sollte Gegenstand weiterer Forschung sein.

Der Einsatzgrund „Hausnotruf aktiver Alarm“ weist die Besonderheit auf, dass die Weitergabe an die ILS erst nach Zwischenschaltung einer Hausnotrufzentrale einer Hilfsorganisation oder eines privaten Betreibers erfolgt. Vor dem Hintergrund dieser zusätzlichen Notrufabfrage werfen die hohen RoT-Quoten bei diesem Einsatzgrund zusätzliche Fragen auf, die im Rahmen dieser Arbeit nicht beantwortet werden können. Verbesserungspotenzial könnte hier zum Beispiel in einem funktionierenden Hintergrunddienst des Hausnotrufbetreibers liegen. Bei dem Hintergrunddienst handelt es sich um eigenständige mobile Einheiten, die vom Hausnotrufbetreiber in eigener Verantwortung ohne Inanspruchnahme des öffentlich-rechtlichen Rettungsdienstes entsendet werden können. Die Leitstellenbereiche der größten bayerischen Städte (ILS München, ILS Nürnberg, ILS Augsburg) wiesen wesentlich niedrigere RoT-Quoten bei dem Einsatzgrund „Hausnotruf aktiver Alarm“ auf als die überwiegend ländlich geprägten Bereiche Coburg, Bayreuth/Kulmbach und Hof. Dies könnte nach Meinung der Autoren ein Indiz dafür sein, dass ein effizienter Hintergrunddienst für einen Hausnotrufbetreiber erst ab einer gewissen Anschlusszahl ökonomisch darstellbar ist. Die lokalen Unterschiede in den RoT-Quoten spiegeln vermutlich unterschiedliche Alarmierungsplanungen sowie differierende Alarmierungsgrundsätze wider. Außerdem könnten unterschiedliche regionale Vereinbarungen zur Zusammenarbeit zwischen Hausnotrufzentralen und Leitstellenbetreibern bestehen.

Wir haben in dieser Arbeit RoT als Indikator für eine möglicherweise nicht gerechtfertigte Inanspruchnahme des öffentlich-rechtlichen Rettungsdienstes gewertet. Pekanoja et al. kommen zu dem Ergebnis, dass bei Nicht-Transport-Situationen oftmals Tätigkeiten durch das Rettungsdienstpersonal übernommen werden, die eigentlich in das Aufgabenfeld anderer Akteure des Gesundheitswesens (z. B. Pflegedienst) fallen [12]. Ebben et al. stellen in einer Übersichtsarbeit basierend auf 67 Studien dar, dass 6,1 % der nicht-transportierten Patienten innerhalb von 24 h erneut einen Rettungsdienstkontakt haben [13].

Während im Bereich des Rettungsdienstes in Bayern die oberste Rettungsdienstbehörde am BayStMI verbindliche Vorgaben für die Alarmierungsplanung erlässt, verbleibt die Festlegung für die Feuerwehralarmierung auf kommunaler Ebene. Dies stellt einen Erklärungsansatz für die regional aufgetretenen Unterscheidungen dar, da insbesondere beim Einsatzgrund mit der höchsten Spannweite („Brandmeldeanlage“) keine Notrufabfrage in der Leitstelle stattfindet und somit jede Kreisverwaltungsbehörde in ihrer regionalen Alarmierungsplanung über den Einsatz eines RTW bei diesem Einsatzgrund entscheidet. Unsere Analyse fokussiert auf die Inanspruchnahme des Rettungsdienstes und unterscheidet nicht zwischen Einsätzen des Rettungsdienstes mit direktem Patientenkontakt und Einsätzen zu Abstellungen für die Feuerwehr.

Die hohen Spannweiten bei den Einsatzgründen „Herz/Kreislauf“ und „Trauma“ weisen auf unterschiedliche Entscheidungen im Rahmen der Notrufabfrage hin. Gerade bei diesen Einsatzgründen könnte es aufgrund subjektiver Bewertung der Notfallschilderung zu unterschiedlichen Dispositionsentscheidungen in Bezug auf den Notarzt kommen. Zur objektiven Einsatzmittelentscheidung sowie zur Erhöhung der Handlungssicherheit der Disponierenden in den ILS sollte über die landesweit verpflichtende Einführung einer standardisierten oder strukturierten Notrufabfrage mit Softwareunterstützung diskutiert werden, um unnötige Alarmierungen mit Fehldispositionen zu vermeiden. Mehrere Veröffentlichungen zeigen positive Effekte der Verwendung von Notrufabfrageprotokollen [14–19].

Ein möglicher weiterer Lösungsansatz könnte sein, bei RoT künftig statt eines RTW alternative Rettungsmittel indikationsbezogen zu entsenden. Ein solches Konzept ist beispielsweise das im Oldenburger Land durchgeführte Projekt „Gemeindenotfallsanitäter“ [20–22] oder das Pilotprojekt „Rettungseinsatzfahrzeug in Bayern“ [23, 24]. Durch die gezielte Alarmierung der neu geschaffenen Ressource Gemeindenotfallsanitäter können Patienten ambulant vor Ort versorgt werden und folglich die Rettungsdienste sowie die Institutionen der Notfallversorgung entlastet werden [20–22]. Mit Ausnahme lokaler Aktivitäten, wie zum Beispiel in Regensburg, sind in Bayern flächendeckend keine alternativen Einsatzmittel vorhanden. Deshalb haben Disponenten in den Leitstellen oftmals keine Alternativen und entscheiden sich mutmaßlich bewusst zur Entsendung eines RTW auch bei Einsatzmeldungen, die keinen Patiententransport vermuten lassen. Zur Optimierung des Gesamtsystems braucht deshalb das Leitstellenpersonal mehr Steuerungspotenzial. In der vorliegenden Studie wurde nicht erforscht, ob alternative Angebote helfen würden, RoT-Ereignisse mit einem anderen Angebot als mit einem RTW abzuwickeln. Hier ist weitere Forschungsarbeit nötig.

Die ambulante Versorgung von Patienten durch den Rettungsdienst in Deutschland war bereits Gegenstand mehrerer Studien [25–27] und zeigte rechtliche Herausforderungen sowie die Notwendigkeit einer besseren sektorenübergreifenden Verzahnung auf. Internationale Studien beschreiben, dass es bei bis zu 30 % der Rettungsdiensteinsätze zu keinem Patiententransport kommt [28–30]. Hoikka et al. wiesen in einer Studie in Nordfinnland nach, dass der überwiegende Teil nichttransportierter Patienten keine weitere medizinische Versorgung bzw. keine Behandlung in einer Notaufnahme benötigt [31]. Günther et al. zeigten in ihrer Untersuchung von ambulanten Kontakten mit dem Rettungsdienst Forschungsbedarf auf, um die Beteiligung des Rettungsdienstes an der ambulanten Notfallversorgung darzustellen und ggf. entsprechend lenken zu können [22, 32].

Einige (notfall-)medizinische Studien analysieren, ob für das Outcome bei bestimmten Erkrankungen die Uhrzeit des Ereigniseintrittes relevant ist [33–35]. Aus diesem Grund wurden in unserer Studie die Häufigkeit von RoT und TP in Abhängigkeit von der Uhrzeit des Einsatzbeginns analysiert. Aufgrund bestehender Diskussionen um eine Ausweitung der Sprechzeiten von Ärzten auf das Wochenende [36, 37] sowie vor dem Hintergrund des Einflusses des Wochentags auf Morbidität und Mortalität von Patienten [38] wurden RoT und TP auch in Abhängigkeit des Wochentages betrachtet. Die Abweichungen in der Transportquote zwischen 7 und 11 Uhr könnten darauf hindeuten, dass sich die Rettungsdienstmitarbeitenden um diese Uhrzeiten aufgrund hohen Einsatzaufkommens für weniger Vor-Ort-Behandlungszeit zugunsten eines zügigen Transportes entscheiden. Denkbar ist auch, dass aufgrund der Öffnung der Arztpraxen zu diesen Zeiten qualifiziertere Notfallmeldungen mit einer ärztlichen Transporteinschätzung an die ILS herangetragen werden, d. h., die Option zum Nicht-Transport ist aufgrund ärztlicher Anordnung gar nicht gegeben. Konträr hierzu ist zu sehen, dass im Rahmen dieser Studie bei der Analyse der Wochentage des Einsatzbeginns Unterschiede auch montags festgestellt werden konnten. Zu diesen Diskussionspunkten sind somit weitere Forschungsarbeiten notwendig.

Für die Häufigkeitsverteilung der RoT-Einsätze in Abhängigkeit vom Wochentag ergeben sich augenscheinlich keine Unterschiede (Abb. 5). Der Chi^2^-Test jedoch zeigt höhere RoT-Quoten für den Montag sowie für das Wochenende. Dieser Unterschied liegt in absoluten Zahlen zwischen 738 bis 823 Einsätzen. Bei einem Mittelwert von 330 Einsätzen für die anderen Tage stellt sich die Frage, ob diese statistische Signifikanz von planerischer Relevanz ist.

Nachts wird in Bayern die Krankentransportkapazität abgesenkt. Eine mögliche Ursache für die erhöhte Transportquote kann auch die vermehrte Kreuzverwendung von RTW für Krankentransporte sein, die in der Nacht z. B. vom ärztlichen Bereitschaftsdienst angeordnet werden.

Laukkanen et al. erforschten, dass das Rettungsdienstpersonal in der Regel in der Lage ist, die Patienten am Einsatzort sicher zu beurteilen [39]. Ein RoT-Einsatz ist auch deshalb nicht gleichbedeutend mit einem nicht notwendigen Einsatz (Fehleinsatz).

Schehadat et al. beschreiben in einer vergleichbaren Analyse monetäre Fehlanreize durch Abrechnungssysteme, bei denen eine Vergütung für den Durchführenden nur bei einem Patiententransport erfolgt [40]. Dieser Fehlanreiz ist in Bayern nicht gegeben, da hier die Finanzierung des Rettungsdienstes vorhaltebezogen mit einer landesweit einheitlichen Abrechnung der Benutzungsentgelte über die mit dem Vollzug gesetzlich beauftragte Zentrale Abrechnungsstelle für den Rettungsdienst Bayern GmbH (ZAST) erfolgt [7].

Leitstellen spielen eine zentrale Rolle im Gesundheitssystem und in der Gefahrenabwehr [41]. Die Leitstelle der Zukunft muss unter Ausnutzung moderner Informationstechnologien und standardisierter Verfahrensabläufe neue Kombinationen von Fähigkeiten für eine flexiblere, effizientere und effektivere Einsatzführung im Fokus haben [42]. Basierend auf den Ergebnissen unserer Studie gehört hierzu auch, dass die Entsendung von RTW zu bestimmten Einsatzgründen hinterfragt und Möglichkeiten zur Implementierung von alternativen Einsatzmitteln zur Entlastung der öffentlich-rechtlichen RTW-Vorhaltung in Erwägung gezogen werden sollten. Das Einsatzfahrtaufkommen im Rettungsdienst hat sich zwischen den Jahren 1994 und 2013 fast verdoppelt [43]. Diesem Trend kann nach Ansicht der Autoren begegnet werden, indem jeder Prozessschritt auf verborgene Ressourcen sowie auf Möglichkeiten zur flexibleren Einsatzführung überprüft wird.

### Limitationen

Disponenten können im Einsatzleitrechner den Einsatzgrund im Einsatzverlauf ändern. Stellt sich der Alarm der Brandmeldeanlage als reales Brandereignis heraus, so kann der Disponent den Einsatzgrund von „Brandmeldeanlage“ (BMA) auf „Brand mit RD“ ändern. Ob und in welchem Umfang solche Anpassungen erfolgen, ist nicht bekannt.

Die Datenbasis sowie die Schlussfolgerungen dieser Studie fußen auf Datenerfassungen der ILS in Bayern sowie bayerischer Gesetzgebung. Eine Übertragung auf andere Bundesländer der Bundesrepublik Deutschland ist aufgrund des vorhaltebezogenen Finanzierungssystems in Bayern nicht ohne Weiteres möglich. Gerade unter diesem Aspekt werden die Studienergebnisse unter anderen Rahmenbedingungen differieren, was aber gerade auch die Relevanz in einem sich ändernden (berufs-)politischen Umfeld betont.

Bei der Interpretation der Ergebnisse muss Beachtung finden, dass die höchsten Spannweiten bei Einsatzgründen mit relativ niedrigen Einsatzzahlen auftraten: „Brandmeldeanlage“ sowie „Hausnotruf aktiver Alarm“.

Eine methodische Limitierung ergibt sich aus der Tatsache, dass bei sämtlichen Einsatzgründen die Disponenten auch die real vorliegenden Zustandsbeschreibungen und nicht die grundsätzlichen Einsatzgründe verwenden könnten. So kann zum Beispiel ein Disponent bei der Meldung einer Hausnotrufzentrale mit dem Meldebild „gestürzte Person“ entscheiden, ob er den Einsatzgrund „Hausnotruf“ oder den Einsatzgrund „Sturz“ wählt. Für eine weitere Bewertung in künftigen Studien muss daher der komplette Einsatzverlauf in der Leitstelle Beachtung finden.

Eine Aussage, ob der jeweilige Notruf direkt von Betroffenen, von unbekannten Dritten oder über eine Vermittlung abgesetzt wird, können wir aus unseren Daten nicht treffen. Die vorliegende Studie gibt zudem keinen Hinweis darauf, weshalb ein Patient nicht transportiert wurde. Hierzu sind weitere Untersuchungen, wie zum Beispiel Befragungen des Rettungsdienstpersonals oder eine Analyse der Einsatzdokumentation, notwendig. Ferner wurde in der vorliegenden Studie die medizinische Sinnhaftigkeit eines Transportes nicht beleuchtet.

Alarmierungsvorgaben können aufgrund regionaler Gegebenheiten oder Vorgaben dazu führen, dass in manchen Leitstellenbereichen bestimmte Einsatzgründe initial mit einem Notarzt beschickt werden. Diese Einsatzgründe könnten in manchen Leitstellenbereichen aufgrund der Ausschlusskriterien dieser Studie unberücksichtigt geblieben sein, da hier nur Einsätze ohne Notarztbeteiligung eingeschlossen wurden. Der Notarztanteil in Bayern liegt im Mittel bei 40 %. Deutliche Unterschiede bestehen zwischen Städten (38 %) und Landkreisen (45 %). Der geringste Notarztanteil in städtischen Bereichen ist in München (22 %), der höchste in Straubing (46 %) dokumentiert. In den Landkreisen differiert der Notarztanteil zwischen 26 % im Landkreis München-Land und 52 % im Landkreis Altötting. Lokale Unterschiede in der Notarztalarmierung könnten einen Selektions-Bias bewirkt haben [2].

Für die vorliegende Studie wurden 45.640 Fälle aufgrund des Vorliegens von lediglich einer bzw. keiner Statusmeldung ausgeschlossen. Dies geschah, um Test- oder Umdispositionen auszuschließen. Die Autoren vermuten jedoch, dass bei einigen der ausgeschlossenen Fälle technische Ursachen oder menschliches Versagen für das Fehlen von Statusmeldungen verantwortlich gewesen sein könnten.

Die vorliegende Studie beleuchtet ausschließlich die Perspektive der ILS. Systemische Veränderungen des Rettungsdienstes, wie zum Beispiel die Einführung des Berufsbildes Notfallsanitäter und die damit ggf. verbundenen Auswirkungen auf die Transportquote, müssen in Folgestudien erforscht werden.

### Schlussfolgerung

Die Studie zeigt regionale und tageszeitliche Unterschiede in der Rate von RoT-Ereignissen. Insbesondere feuerwehrbezogene Rettungsdiensteinsätze und Hausnotrufereignisse tragen zu hohen RoT-Raten bei. Hier finden sich strukturelle Unterschiede zwischen den Leitstellenbereichen. Die Vorhaltung eigener Hintergrunddienste der Hausnotrufbetreiber könnte hier beispielsweise helfen, die Anzahl an Hilfeleistungen durch RTW zu reduzieren. In Zusammenschau kommen wir zu der Schlussfolgerung, dass der Alarmierungsplanung eine erhebliche Bedeutung für die Ressourcenauslastung des öffentlich-rechtlichen Rettungsdienstes zukommt. Ein Nicht-Transport (RoT) ist zwar nicht gleichbedeutend mit einem Fehleinsatz, jedoch könnten alternative Einsatzmittel ebenfalls zu einer Verringerung der Auslastung von RTW führen. Somit hat die Alarmierungsplanung Einfluss auf die Auslastung der verfügbaren Ressourcen und kann künftig bei Vorhandensein von alternativen Einsatzmitteln dazu beitragen, eine weitere Zunahme der RTW-Vorhaltung zu verhindern. Weitere Forschung ist nötig, um die verborgenen Ressourcen in Zukunft besser nutzen zu können.

## Supplementary Information




