# Supplementary material for: Differences between emergency medical services operations with and without patient transport: A retrospective analysis of dispatch center data in a territorial state
Source: Bundesgesundheitsblatt Gesundheitsforschung Gesundheitsschutz. 2022 Sep 16;65(10):996–1006. [Article in German] doi: 10.1007/s00103-022-03590-3 (PMC9522751; doi:10.1007/s00103-022-03590-3)
Supplement: Supplementary file 1 [file 103_2022_3590_MOESM1_ESM.pdf]

Bundesgesundheitsblatt – Gesundheitsforschung – Gesundheitsschutz, Ausgabe 10/2022

Onlinematerial zum Beitrag:

Unterschiede zwischen Rettungsdiensteinsätzen mit und ohne Patiententransport. Eine retrospektive Analyse der Leitstellendaten in einem Flächenstaat

Florian Dax[1,3], Heiko Trentzsch[1], Marc Lazarovici[1], Kathrin Hegenberg[1], Katharina Kneißl[1], Florian Hoffmann[2], Stephan Prückner[1]

[1] Institut für Notfallmedizin und Medizinmanagement (INM), Klinikum der Universität München, LMU München, München, Deutschland
[2] Dr. von Haunersches Kinderspital, Kinderklinik und Kinderpoliklinik, Klinikum der Universität München, LMU München, München, Deutschland
[3] Bayerisches Rotes Kreuz (BRK), Landesgeschäftsstelle, München, Deutschland

Korrespondenzadresse:

Priv.-Doz. Dr. Stephan Prückner
Institut für Notfallmedizin und Medizinmanagement (INM)
Klinikum der Universität München
LMU München
Schillerstr. 53
80336 München
Deutschland
gs.inm@med.uni-muenchen.de

Inhalt:

Tabelle 1: Definitionen der 18 Einsatzgründe mit 1.000 oder mehr Einsätzen im Jahr 2018

Tabelle Z1: Definitionen der 18 Einsatzgründe mit 1.000 oder mehr Einsätzen im Jahr 2018.

Quelle: Bayerisches Staatsministerium des Innern, für Sport und Integration (12.07.2016): Alarmierung im Rettungsdienst, Brand- und Katastrophenschutz in Bayern. Alarmierungsbekanntmachung (ABek) Fundstelle: AllMBl. 2016 S. 1575 [6]

Abkürzungen: ÄDB = Ärztlicher Bereitschaftsdienst, RD = Rettungsdienst, RTW = Rettungswagen

Einsatzgrund	Definition
BMA (Brandmeldeanlage)	*Automatisch ausgelöste Brand- oder Rauchwarnanlage mit Entsendung mindestens eines RTW*
Brand mit RD – mit und ohne vitale Bedrohung	*Brandereignis mit Entsendung mindestens eines RTW*
Hausnotruf aktiver Alarm	*Einsatzweitergabe durch die Hausnotrufzentrale bei aktiven Alarmen des Hausnotrufs (zum Beispiel Betätigung des Notrufknopfs für Senioren)*
Technische Hilfeleistung (THL) mit Rettungsdienst	*Technische Hilfeleistung der Feuerwehr mit Entsendung mindestens eines RTW (zum Beispiel bei eingeschlossenen Personen)*
Kind - (bis 12 Jahre) erkrankt	*akute Symptomatik, die nicht an den Hausarzt oder ÄBD verwiesen werden kann, z. B. Ingestion von potenziell toxischen Substanzen ohne klinische Symptomatik*
Intoxikation	*Intoxikation mit potenziell giftigen Substanzen ohne Hinweis auf eine vitale Störung von Herz, Kreislauf oder Bewusstsein*
Ärger	*verletzt nach Schlägerei ohne akute Vitalbedrohung*
Psych	*psychiatrischer Zustand, der den Verweis an den Hausarzt bzw. ÄBD nicht zulässt*
Bewusstsein	*neu aufgetretene, nicht zunehmende Bewusstseinsstörung unter Ausschluss einer vitalen Indikation, die nicht an den Hausarzt oder ÄBD verwiesen werden kann*
Kind (bis 12 Jahre) Trauma	*akute Verletzungen mit der Notwendigkeit einer zeitnahen Versorgung ohne Anhalt für vitale Gefährdung unter Berücksichtigung des Unfallmechanismus*
Trauma - Verkehrsunfall (VU) nur Rettungsdienst	*akute Verletzungen mit der Notwendigkeit einer zeitnahen Versorgung ohne Anhalt für vitale Gefährdung unter Berücksichtigung des Unfallmechanismus*
Sonstiges Ereignis/Zustand	*Stoffwechselentgleisung ohne klinische Symptomatik, gastrointestinale oder gynäkologische Blutung, Nasenbluten, Entgleisungen der Körpertemperatur*
Herz/Kreislauf	*neu aufgetretene, nicht zunehmende Herz- oder Kreislaufbeschwerden unter Ausschluss einer vitalen Indikation, die nicht an den Hausarzt oder ÄBD verwiesen werden können*
Atmung	*neu aufgetretene, nicht zunehmende Atembeschwerden unter Ausschluss einer vitalen Indikation, die nicht an den Hausarzt bzw. ÄBD verwiesen werden kann*
Neuro	*neu aufgetretene, nicht zunehmende neurologische Ausfälle (z. B. Schlaganfallsymptome) ohne Bewusstseinsstörung, Zustand nach einmaligem Krampfanfall, sonstiger neurologischer Zustand, der einen Verweis an den Hausarzt bzw. ÄBD nicht zulässt*
Schmerzen	*neu aufgetretene, nicht zunehmende Schmerzen, die den Verweis an den Hausarzt bzw. den ÄBD nicht zulassen*
Trauma	*akute Verletzungen mit der Notwendigkeit einer zeitnahen Versorgung ohne Anhalt für vitale Gefährdung unter Berücksichtigung des Unfallmechanismus, Stromunfall ohne Symptomatik*
Geburt/Entbindung	*Wehentätigkeit, Abgang Fruchtwasser, Geburt nicht unmittelbar bevorstehend*
